# Mechanism Prediction of *Astragalus membranaceus* against Cisplatin-Induced Kidney Damage by Network Pharmacology and Molecular Docking

**DOI:** 10.1155/2021/9516726

**Published:** 2021-08-19

**Authors:** Congchao Jia, Xianchao Pan, Binyou Wang, Pengyu Wang, Yiwei Wang, Rong Chen

**Affiliations:** ^1^Clinical Medical College, Southwest Medical University, Luzhou, Sichuan 646000, China; ^2^Department of Medicinal Chemistry, College of Pharmacy, Southwest Medical University, Luzhou, Sichuan 646000, China; ^3^College of Pharmacy, Southwest Medical University, Luzhou, Sichuan 646000, China; ^4^State Key Laboratory of Biotherapy, West China Hospital, Sichuan University, Chengdu 610041, China; ^5^College of Basic Medical Sciences, Southwest Medical University, Luzhou 646000, China; ^6^Department of Pathophysiology, College of Basic Medical Sciences, Southwest Medical University, Luzhou 646000, China

## Abstract

**Background:**

Cisplatin is a frequently used and effective chemotherapy drug in clinical practice, but severe side effects limit its use, among which nephrotoxicity is considered the most serious and prolonged damage to the body. *Astragalus membranaceus* (AM) is a well-known herbal medicine, and modern pharmacological studies have confirmed its antioxidant, immunomodulatory, and antiapoptotic effects. Clinical studies have shown that AM and its active components can attenuate cisplatin-induced kidney damage, but the molecular mechanism has not been fully expounded.

**Materials and Methods:**

First, the components and targets information of AM were collected from the TCMSP, and the relevant targets of cisplatin-induced kidney damage were accessed from the GeneCards and OMIM databases. Then, the core targets were selected by the Venn diagram and network topology analysis, which was followed by GO and KEGG pathway enrichment analysis. Finally, we construct a component-target-pathway network. Furthermore, molecular docking was carried out to identify the binding activity between active components and key targets.

**Results:**

A total of 20 active components and 200 targets of AM and 646 targets related to cisplatin-induced kidney damage were obtained. 91 intersection targets were found between AM and cisplatin-induced kidney damage. Then, 16 core targets were identified, such as MAPK1, TNF-*α*, and p53. Furthermore, GO and KEGG pathway enrichment analysis suggested that MAPK, Toll-like receptor, and PI3K-Akt signaling pathways may be of significance in the treatment of cisplatin-induced kidney damage by AM. Molecular docking indicated that quercetin and kaempferol had high binding affinities with many core targets.

**Conclusion:**

In summary, the active components, key targets, and signaling pathways of AM in the treatment of cisplatin-induced kidney damage were predicted in this study, which contributed to the development and application of AM.

## 1. Introduction

Cisplatin, a heavy metallic compound with powerful anticancer effects, is synergistic with many antitumor drugs without cross-resistance, thus being one of the most frequently used and effective chemotherapy drugs at present [1]. However, it has many serious side effects that greatly limit its therapeutic use, including gastric toxicity, ototoxicity, nephrotoxicity, allergic reactions, and bone marrow suppression [2]. Nephrotoxicity of cisplatin is considered the most serious and prolonged damage to the body [3]. Acute kidney injury (AKI) has been reported in 25–30% of patients roughly, who have received cisplatin chemotherapy [4]. The mechanisms of cisplatin-induced kidney damage are very complicated, involving multiple factors such as oxidative damage, dysfunctional mitochondria, and renal tubular epithelial cell apoptosis [5–9]. Although hydration diuresis and diuretic forced diuresis are commonly used clinically to reduce cisplatin-induced kidney damage, the results are still unsatisfactory [10, 11]. Therefore, there is an immediate need to pursue an effective measure to mitigate cisplatin-induced kidney damage.

*Astragalus membranaceus* (AM), also known as “Huang Qi” in traditional Chinese medicine, is the dried root of membranous Astragalus or Astragalus mongolicus, which has been widely used in the clinical setting [12]. AM, which has a long history in China, was first recorded in *Shennong*'*s Herbal Classic* (an ancient classic Chinese herbal medicine book). Modern pharmacological studies have shown that AM contains flavonoids, saponins, and other active ingredients, which have a wide range of pharmacological effects, such as antioxidant, immune regulation, antiapoptosis, and antitumor [13–16]. A recent study indicated that Astragalus polysaccharide (an active ingredient of AM) could reduce AKI induced by cisplatin through protecting mitochondria, inhibiting oxidative damage, and improving mitochondria-mediated apoptosis [17]. Also, according to a meta-analysis, AM injection can alleviate the clinical side effects of platinum-based chemotherapy drugs [18]. However, the targets and specific mechanism of AM against cisplatin-induced kidney damage remain largely unknown.

**Figure 1:**
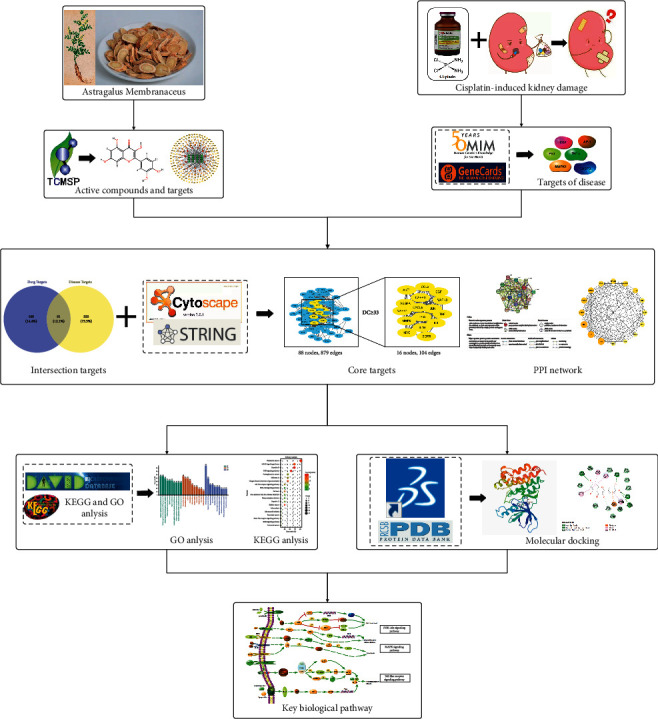
The workflow of this study.

Recently, network pharmacology has been a potent tool for gaining insights into the mechanisms of drugs [19]. This method explores the drug-component-target-pathway-disease relationship from a systems biology perspective, predicts potential drug treatment mechanisms for diseases, and provides a theoretical basis for the mechanisms of Chinese medicine treatment for diseases [20]. Molecular docking is an efficient computational method for the prediction of the binding affinity between a ligand and a target protein, which can be used to identify potential drug targets [21]. In this paper, the possible targets and the molecular mechanism of AM on the treatment of cisplatin-induced kidney damage were investigated by network pharmacology and molecular docking (Figure 1).

## 2. Materials and Methods

### 2.1. Screening Main Active Components and Targets of AM

Information of the active components and targets associated with AM was collected from the TCMSP database (https://tcmspw.com/, February 20, 2021) [22]. The TCMSP database is a systematized pharmacology resource that provides the ADME (absorption, distribution, metabolism, and excretion) characteristics of Chinese herbal medicines or ingredients including oral bioavailability (OB), drug likeness (DL), blood-brain barrier, Caco-2 permeability, etc. [23]. Among the pharmacokinetic properties mentioned above, OB and DL are the most vital ones for drug delivery, as they help assess the effect of drug distribution in the circulatory system and how drug-like compounds regard factors such as bioavailability [24]. In the TCMSP database, OB is based on the internal model OBioavail 1.1 and DL is evaluated according to molecular descriptors and Tanimoto coefficients [22, 25]. In this study, the name of the herb “Huang Qi” was inputted into the searching box to get the active components and related targets. We selected active components with OB ≥ 30% and DL ≥ 0.18 and their corresponding targets. All targets associated with these active ingredients (both validated and predicted targets) were then imported into the UniProt database (https://www.uniprot.org/, February 15, 2021) to get the gene symbol of targets.

### 2.2. Targets Collection of Cisplatin-Induced Kidney Damage

By using the keywords of “cisplatin-induced kidney damage,” “cisplatin-induced nephrotoxicity,” “cisplatin kidney damage,” and “cisplatin-induced kidney injury,” gene symbols of disease-related targets were obtained from the OMIM (https://omim.org) and the GeneCards (https://www.genecards.org) databases [26–28]. The results accessed from GeneCards were screened for the relevance score ≥10.00 as the screening index. We chose Gene Map in “Advanced Search” to search target genes related to disease in the OMIM database. The ultimate disease-related targets were obtained by removing the duplicates.

### 2.3. Screening Core Targets of AM in Cisplatin-Induced Kidney Damage Treatment

The VENNY 2.1 online platform (https://bioinfogp.cnb.csic.es/tools/venny/) was used for intersection targets between AM and cisplatin-induced kidney damage. The intersection targets were used to constitute the protein-protein interaction (PPI) network using the STRING database (https://string-db.org/, February 28, 2021) [29] with confidence scores larger than 0.7, and the other variables were left at their original values. We then exported the PPI network in TSV format and analyzed its topology properties (degree centrality) using Cytoscape 3.8.0 (https://cytoscape.org/) [30]. Degree centrality (DC) is the most commonly used topological parameter, which is utilized to appraise the central attribute of nodes in the network. The targets with the DC ≥ 2 × median were selected as the core targets.

### 2.4. Constructing and Analyzing PPI Network of Core Targets

The core targets obtained above were inputted into the STRING database to get the PPI network. All settings were the same as above. Then, the PPI network was extracted in TSV format and inputted into Cytoscape 3.8.0 for further analysis.

### 2.5. Functional and Pathway Enrichment Analysis of Core Targets

To investigate the multiple mechanisms of AM against cisplatin-induced kidney damage at the systematic level further, we used DAVID 6.8 (https://david.ncifcrf.gov/, March 5, 2021) [31] to perform the functional enrichment analysis of core targets, including the Gene Ontology (GO) terms as well as Kyoto Encyclopedia of Genes and Genomes (KEGG) pathways [32, 33].

### 2.6. AM Active Component-Target-Pathway Analysis

To investigate the relationship between active components, targets, and pathways, we constructed the network of active component-target-pathway (C-T-P network). We imported active components, targets, and pathways into Cytoscape 3.8.0 as three types of nodes and connected associated nodes with edges to construct the active components-targets-pathways network.

### 2.7. Molecular Docking

We used molecular docking to predict the binding activity between the active ingredient and the core target. The structural formula of active components was obtained from PubChem (https://pubchem.ncbi.nlm.nih.gov/, March 10, 2021) and stored in SDF format. Afterward, the core target conformations were obtained from the Protein Data Bank database (PDB, https://www.rcsb.org/, March 10, 2021). The following were the screening conditions: (1) The biological source of protein structure is human; (2) The protein structure is acquired by X-crystal diffraction; (3) The resolution of protein crystals is less than 3 Å; and (4) Protein structures with unique ligands are preferred. The structures of the ligands and the protein receptors were prepared by using Discovery Studio (DS, BIOVIA, 2019). Then, we used the flexible docking procedure CDOCKER built-in DS for molecular docking with CHARMM force field (Chemistry at HARvard Macromolecular Mechanics).

### 2.8. Database and Software Summary

The databases and software used in this study are listed in Table 1 for the convenience of readers.

## 3. Results

### 3.1. Active Components and Targets of AM

**Figure 2:**
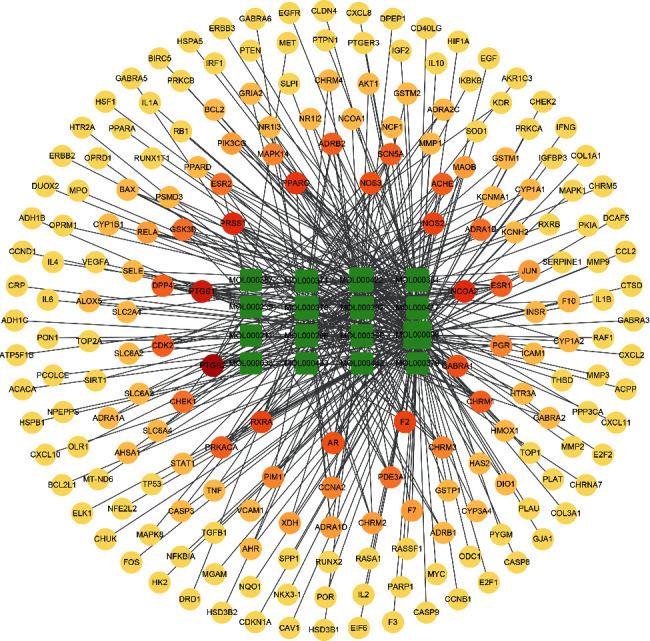
Network of active component-target. The green square means the active components of AM, and its DC value is represented by the node size. The circular node means the active components' targets, and a darker node color indicates a larger degree value. The interaction between the components and the targets is represented by the edge.

We gained 86 components from the TCMSP database, mainly including quercetin, isoflavanone, kaempferol, and so forth. A total of 20 active components satisfied the criteria of OB ≥ 30% and DL ≥ 0.18 (Table 2). Then, we obtained a total of 200 targets related to these 20 active components from TCMSP.  Figure 2 shows the interaction between active components and targets. It could be seen that quercetin had the maximal DC, that is to say, quercetin had the most targets, suggesting that quercetin might be an important active component of AM.

### 3.2. Targets Related to Cisplatin-Induced Kidney Damage

We found 534 relevant targets of cisplatin-induced kidney damage in GeneCards and 121 targets in OMIM databases. After deleting duplicate targets, 646 disease targets were finally obtained. The top 5 targets for relevance score were cellular tumor antigen p53 (p53), tumor necrosis factor-alpha (TNF-*α*), interleukin-6 (IL-6), angiotensin-converting enzyme, and transforming growth factor-beta 1.

### 3.3. Core Targets of AM in Cisplatin-Induced Kidney Damage Treatment

**Figure 3:**
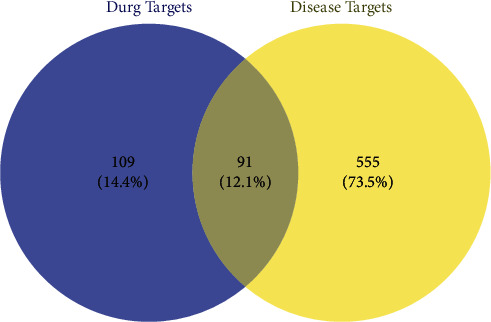
Intersection targets of AM and cisplatin-induced kidney damage. The blue zone indicates targets for active components of AM; the yellow zone indicates targets for cisplatin-induced kidney damage; and the overlap zone indicates the intersection targets.

**Figure 4:**
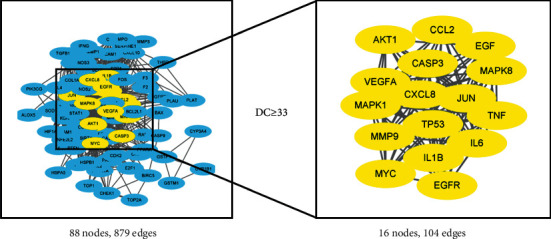
The process of filtering core targets. 16 core targets with DC ≥ 33 (right panel) were selected from 91 intersection targets (left panel). Because 3 of the 91 common targets have no interaction with other targets with high confidence (confidence scores >0.7), there are only 88 nodes in the PPI network.

The Venn diagram showed that there were 91 intersection targets of AM and cisplatin-induced kidney damage (Figure 3). The intersection targets were used to construct the PPI network using the STRING data set. Immediately afterward, the network was introduced into Cytoscape 3.8.0 and the DC of each node was calculated by using the function of “network analyzer.” 16 core targets of AM in cisplatin-induced kidney damage treatment were singled out with DC ≥ 33 (2 × 16.5) (Figure 4).  Table 3 shows the specific information of the 16 core targets.

### 3.4. Constructing and Analyzing PPI Network

**Figure 5:**
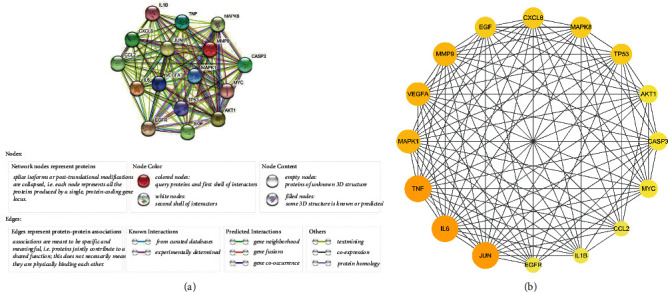
(a) The network from STRING platform directly. Nodes represent core targets; various edges mean different interactions. (b) The network was processed by Cytoscape 3.8.0. The interaction among the core targets is represented by the inner edge. The outer ring is arranged clockwise in descending order of significance of the nodes.

The 16 core targets were introduced into the STRING database to get the PPI network (Figure 5(a)). The DC was reckoned with “Network Analyzer” in Cytoscape 3.8.0 to reflect the significance of a target in the network. Significant targets are indicated by darker colors, larger sizes, and higher DC of interaction in the network, such as mitogen-activated protein kinase 1 (MAPK1), TNF-*α*, IL-6, vascular endothelial growth factor A (VEGFA) (Figure 5(b)).

### 3.5. GO and KEGG Enrichment Analysis

**Figure 6:**
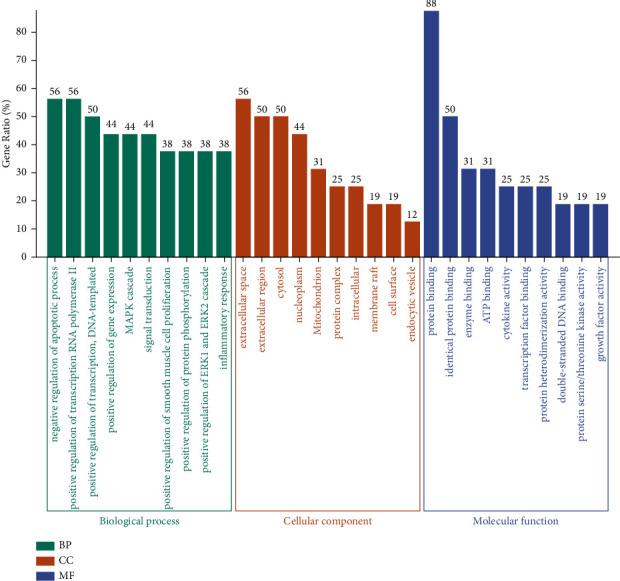
GO enrichment analysis. GO items and gene ratio are represented by the *x*-axis and *y*-axis, respectively.

**Figure 7:**
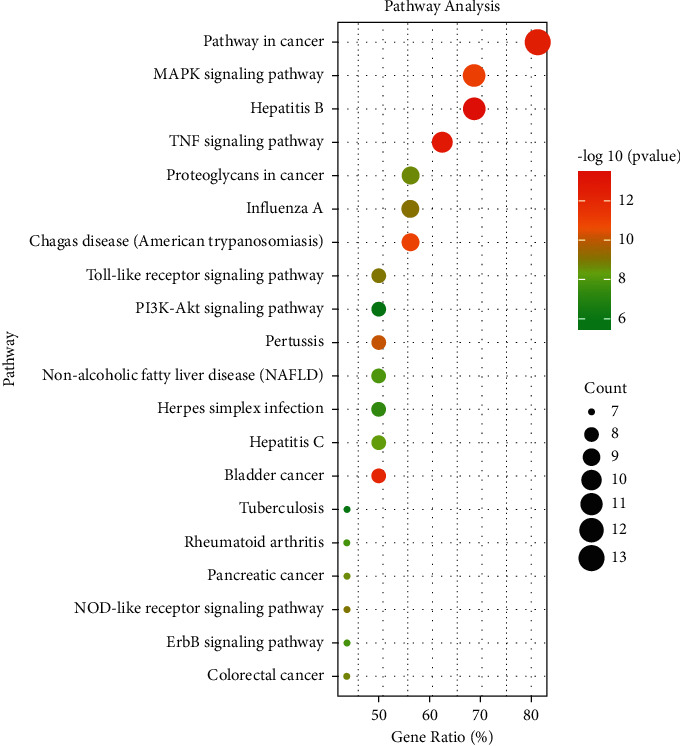
KEGG enrichment analysis. Gene ratio and pathways are represented by the *x*-axis and *y*-axis, respectively; the size and color of the dots indicate the gene count and the level of *P* value, respectively.

For the purpose of figuring out the mechanism of AM against cisplatin-induced kidney damage more systematically, the 16 core targets were inputted into DAVID 6.8 to GO and KEGG enrichment analysis. There were 186 items related to biological processes, mainly including MAPK cascade, positive regulation of smooth muscle cell proliferation, inflammatory response, negative regulation of the apoptotic process, and so on. In the cell composition, the extracellular region is the main classification of these targets. Additionally, there were 23 terms enriched in the molecular function category, mainly including protein serine/threonine kinase activity, cytokine activity, transcription factor binding, growth factor activity, and so on. We selected the top 10 items according to the gene ratio, which are presented in Figure 6. The KEGG enrichment pathways analysis results revealed that there were 91 pathways in total, mainly include MAPK signaling pathway, PI3K-Akt signaling pathway, TNF signaling pathway, etc. We selected the top 20 pathways of KEGG according to the gene ratio, which are presented in Figure 7.

### 3.6. Active Component-Target-Pathway Analysis

**Figure 8:**
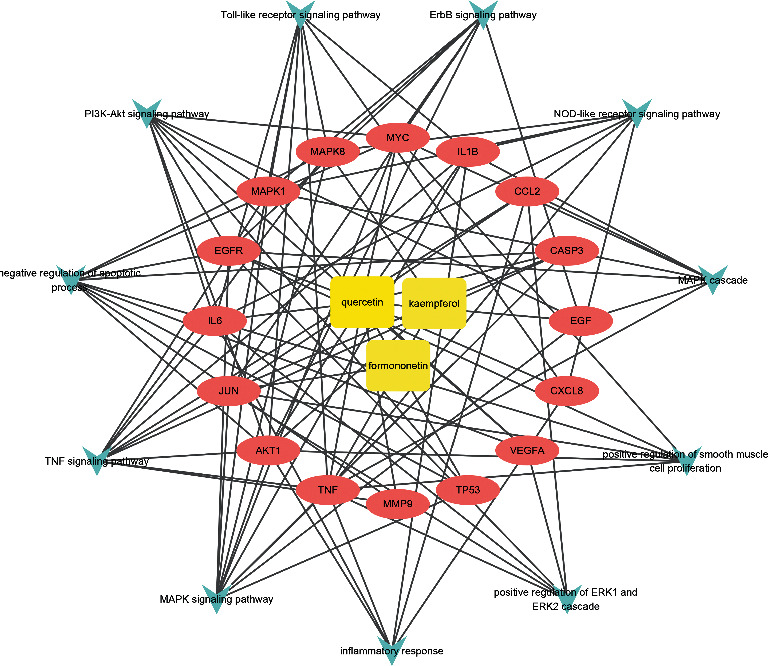
The C-T-P network of AM against cisplatin-induced kidney damage. The significant compounds, core targets, and pathways are represented by the yellow rectangular nodes, pink oval nodes, and arrow nodes, respectively.

For the aim at clarifying what the relationships between components, core targets, and pathways are, the significant pathways and biological processes, core targets, and active components of AM were used to build the C-T-P network (Figure 8). The targets related to pathways and biological processes are also listed in Table 4.

### 3.7. Molecular Docking

To identify potential drug-target interactions, we performed molecular docking to predict the binding affinities between the active components and core targets in the C-T-P network. Docking results are shown in Table 5. Herein, CDOCKER_ENERGY was used to evaluate the binding affinities. Lower binding energy represents a higher affinity between a ligand and a protein [34]. It can be seen that quercetin has a higher affinity with MAPK1, p53, matrix metalloproteinase-9 (MMP-9), epidermal growth factor receptor (EGFR), and caspase-3, suggesting that quercetin may mitigate cisplatin-induced kidney damage by binding to them.

## 4. Discussion

Cisplatin-induced kidney damage is a renal impairment caused by cisplatin, which is extremely harmful to human beings. It is reported that about 25–30% of patients developed symptoms of AKI after receiving cisplatin chemotherapy [4]. Besides hydration combined with mannitol diuretic, injection of AM or its active components is often used for treatment in the clinic [35–37]. Although the anti-cisplatin-induced kidney damage efficacy of AM has been demonstrated, the mechanism has not yet been clarified. Consequently, network pharmacology and molecular docking were performed to analyze the active components, targets, and related signaling pathways of AM against cisplatin-induced kidney damage and to figure out the potential mechanism of AM anti-cisplatin-induced kidney.

We obtained 20 active components and 200 corresponding targets from the TCMSP database and then constructed a component-target network, which mainly included quercetin, formononetin, isorhamnetin, and kaempferol. Modern pharmacological studies showed that quercetin has an anti-cisplatin-induced kidney injury effect, which is related to its antioxidant activity and the ability to inhibit kidney inflammation and renal tubular cell apoptosis [38]. In a recent study, it was reported that pretreatment with kaempferol reduced cisplatin-mediated oxidative stress, apoptosis, inflammation, kidney injury, as well as improved its function [39]. Formononetin, an O-methylated isoflavone, is one of the main bioactive ingredients in red clover plants. It was reported that formononetin can alleviate AKI induced by cisplatin and has many potential pharmacological effects, like anti-inflammatory, antioxidant, and antiapoptosis [40]. Isorhamnetin, a metabolite of quercetin, not only has anti-inflammatory and antioxidant effects but also enhances the anticancer effect of cisplatin [41, 42]. In addition, it is interesting that multiple AM active components can act on multiple different targets and have common pharmacological effects, which embodies the peculiarities of AM multicomponent, multitarget synergistic treatment.

We obtained 91 intersection targets between AM and cisplatin-induced kidney damage and further screened out 16 core targets of AM in the treatment of cisplatin-induced kidney damage. The targets are mainly associated with oxidative stress, apoptosis, inflammation, and cell proliferation. According to quantities of studies, apoptosis induced by TNF-*α*, p53, and caspase-3 [43, 44] and inflammation induced by IL-6, TNF-*α*, and interleukin-1 beta (IL-1*β*) played an essential role in cisplatin-induced kidney damage [45, 46]. It was reported that cisplatin increased the expression of proinflammatory cytokine TNF-*α*, which induced an exogenous apoptotic pathway through its tumor necrosis factor receptor 1 (TNFR1) [47], and TNF-*α* can activate proinflammatory cytokines and chemokines such as NF-*κ*B and trigger oxidative stress, which ultimately aggravates kidney damage [48]. The study by Xu et al. also demonstrated that the expression of TNF-*α* further helped induce the expression of receptor-interacting protein 1 (RIP1), RIP3, and mixed lineage kinase domain-like protein (MLKL) in proximal tubular cells and enhanced the necrotic signaling pathway through positive feedback [49]. As mentioned in a literature review, the role of p53 in cisplatin-induced cytotoxicity mainly involved activation of the mitochondrial apoptotic pathway [47]. After exposure to cisplatin-induced cellular DNA damage, p53 was phosphorylated, and the proapoptotic protein Bax underwent structural modifications and mitochondrial membrane integrity changes, eventually downregulating the antiapoptotic proteins Bcl-2 and Bcl-xL and triggering the mitochondrial apoptotic pathway [50]. In addition, Yuan et al. proved that p53 promotes cisplatin-induced renal oxidative damage and apoptosis by regulating P66shc and manganese-dependent superoxide dismutase (MnSOD) [51]. A recent study showed that the AMPK-p53-Bax signaling pathway played a crucial role in cisplatin-induced apoptosis of renal tubular epithelial cells [44]. Many studies have shown that IL-6 is closely related to the inflammatory response in cisplatin-induced renal injury and cisplatin-induced inflammatory response can be alleviated by reducing the expression of IL-6 [52–55]. Another study showed that IL-6 mediates the production and elimination of ROS in cisplatin-induced AKI [56]. All of the above-mentioned core targets and those not mentioned due to word limits are summarized in Table 6.

**Figure 9:**
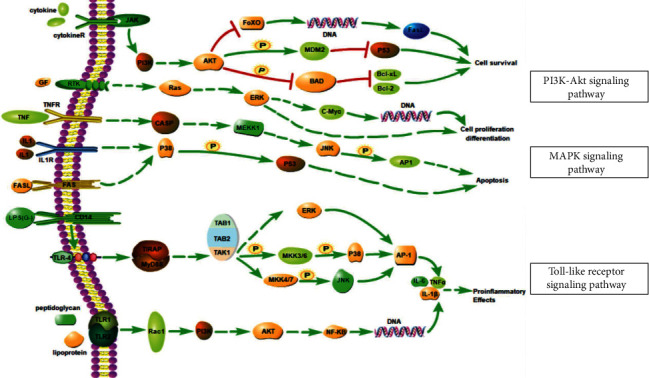
Key biological pathways.

Subsequently, GO and KEGG enrichment analyses were applied to 16 core targets. The result suggested that the PI3K-Akt signaling pathway, MAPK signaling pathway, and Toll-like receptor signaling pathway were suggested to have a significant role in the treatment of cisplatin-induced kidney damage by AM. It has been reported that the MAPK pathway is central to the regulation of inflammation and oxidative stress in cisplatin renal injury and that TNF-*α* can activate the MAPK pathway [65]. Besides, Ning et al. found the PI3K-Akt signaling pathway plays an essential role in defending renal tubular epithelial cells from apoptosis induced by cisplatin [66]. Meanwhile, many studies have demonstrated that quercetin can suppress the formation of reactive oxygen species, activate the JNK/P38 MAPK signaling pathway, and regulate the PI3K-Akt pathway [67–69]. Therefore, quercetin may reduce oxidative stress by regulating the MAPK pathway and PI3K-Akt pathway in the treatment of cisplatin-induced kidney. The results of molecular docking suggested that the most likely key targets are MAPK1, TP53, and EGFR. In addition, kaempferol could block the MAPK cascade and reduce the expression of JNK, TNF-*α*, and ERK1/2, thus improving cisplatin-induced renal injury [39, 70]. What is more, the Toll-like receptor signaling pathway can activate the releasing of downstream cytokines such as TNF-*α* and IL-1*β*, leading to increased inflammatory cell infiltration and inflammatory responses in renal tissue [71, 72]. Meanwhile, a study has shown that kaempferol dramatically suppresses the upregulation of toll-like receptor 4, blocking the Toll-like receptor signaling pathway to reduce the inflammatory response [70]. Molecular docking suggested that quercetin most likely affected some pathways by combining with p53, MAPK, MMP-9, EGFR, and caspase-3 and then finally achieved the effect of reducing cisplatin-induced renal damage. The pathways discussed above are shown in Figure 9.

In the C-T-P network, each pathway corresponds to a plurality of targets, and each target is connected with a plurality of pathways that are connected with each other through a common target. It was suggested that AM synergistically treats cisplatin-induced kidney damage through a multicomponent, multitarget, and multipathway mechanism.

## 5. Conclusions

In summary, the present research aimed to figure out the molecular mechanism of AM against cisplatin-induced kidney damage by network pharmacology and molecular docking. Screening of active ingredients and molecular docking studies showed that quercetin, kaempferol, and formononetin were significant active ingredients to AM against cisplatin-induced kidney damage. The C-T-P network suggested that the MAPK pathway, PI3K-Akt signaling pathway, and Toll-like receptor signaling pathway had important roles in AM treatment of cisplatin-induced kidney damage. In addition, one of the significant findings is that AM treats cisplatin-induced kidney injury to multicomponent, multitarget, and multipathway synergistic effects. Unfortunately, this study has no corresponding experimental validation that is what we need to do in the future. In a nutshell, this study provided a rationale for further research of cisplatin-induced renal injury and a new orientation of development and application of AM.

## Figures and Tables

**Table 1:** Database and software summary.

Names of database and software	Website
Traditional Chinese Medicine Systems Pharmacology (TCMSP) database	https://tcmspw.com/
UniPort database	https://www.uniprot.org/
Online Mendelian Inheritance in Man (OMIM)	https://omim.org/
GeneCards database	https://www.genecards.org/
VENNY 2.1 online platform	https://bioinfogp.cnb.csic.es/tools/venny/
Cytoscape 3.8.0	https://cytoscape.org/
STRING database	https://string-db.org/
DAVID 6.8	https://david.ncifcrf.gov/
PubChem	https://pubchem.ncbi.nlm.nih.gov/
RCSB Protein Data Bank	https://www.rcsb.org/

**Table 2:** Information about active components of AM.

Mol ID	Molecule name	OB (%)	DL	DC
MOL000098	Quercetin	46.43	0.28	144
MOL000422	Kaempferol	41.88	0.24	58
MOL000378	7-O-Methylisomucronulatol	74.69	0.30	43
MOL000392	Formononetin	69.67	0.21	36
MOL000354	Isorhamnetin	49.60	0.31	33
MOL000371	3,9-Di-O-methylnissolin	53.74	0.48	22
MOL000296	Hederagenin	36.91	0.75	20
MOL000380	(6aR,11aR)-9,10-Dimethoxy-6a,11a-dihydro-6H-benzofurano [3,2-c]chromen-3-ol	64.26	0.42	20
MOL000417	Calycosin	47.75	0.24	20
MOL000239	Jaranol	50.83	0.29	11
MOL000387	Bifendate	31.10	0.67	5
MOL000433	FA	68.96	0.71	3
MOL000442	1,7-Dihydroxy-3,9-dimethoxy pterocarpene	39.05	0.48	3
MOL000379	9,10-Dimethoxypterocarpan-3-O-*β*-D-glucoside	36.74	0.92	2
MOL000033	(3S,8S,9S,10R,13R,14S,17R)-10,13-Dimethyl-17-[(2R,5S)-5-propan-2-yloctan-2-yl]-2,3,4,7,8,9,11,12,14,15,16,17-dodecahydro-1H-cyclopenta[a]phenanthren-3-ol	36.23	0.78	1
MOL000211	Mairin	55.38	0.78	1
MOL000374	5′-Hydroxyiso-muronulatol-2′,5′-di-O-glucoside	41.72	0.69	0
MOL000398	Isoflavanone	109.99	0.3	0
MOL000438	(3R)-3-(2-Hydroxy-3,4-dimethoxyphenyl)chroman-7-ol	67.67	0.26	0
MOL000439	Isomucronulatol-7,2′-di-O-glucosiole	49.28	0.62	0

**Table 3:** The specific information of the 16 core targets.

Gene symbol	Protein name	DC
TP53	Cellular tumor antigen p53	56
AKT1	RAC-alpha serine/threonine-protein kinase	53
IL6	Interleukin-6	52
TNF	Tumor necrosis factor-alpha	50
VEGFA	Vascular endothelial growth factor A	47
JUN	Transcription factor AP-1	46
MAPK1	Mitogen-activated protein kinase 1	45
MAPK8	Mitogen-activated protein kinase 8	45
MMP9	Matrix metalloproteinase-9	38
EGF	Pro-epidermal growth factor	37
IL1B	Interleukin-1 beta	37
MYC	Myc proto-oncogene protein	36
EGFR	Epidermal growth factor receptor	35
CXCL8	Interleukin-8	34
CASP3	Caspase-3	34
CCL2	C-C motif chemokine 2	33

**Table 4:** Information of related target gene symbols.

Pathway or biological process	Gene symbol of related target
MAPK signaling pathway	JUN, MAPK8, EGF, IL1B, MYC, CASP3, MAPK1, AKT1, TNF, TP53, EGFR
TNF signaling pathway	IL6, JUN, MAPK8, IL1B, CASP3, MAPK1, CCL2, AKT1, TNF, MMP9
Toll-like receptor signaling pathway	IL6, JUN, MAPK8, CXCL8, IL1B, MAPK1, AKT1, TNF
Positive regulation of smooth muscle cell proliferation	IL6, JUN, MYC, AKT1, TNF, EGFR
PI3K-Akt signaling pathway	IL6, EGF, MYC, MAPK1, AKT1, TP53, EGFR, VEGFA
NOD-like receptor signaling pathway	IL6, MAPK8, CXCL8, IL1B, MAPK1, CCL2, TNF
ErbB signaling pathway	JUN, MAPK8, EGF, MYC, MAPK1, AKT1, EGFR
Negative regulation of apoptotic process	IL6, MAPK8, MYC, CASP3, AKT1, TP53, MMP9, EGFR, VEGFA
MAPK cascade	EGF, IL1B, MYC, MAPK1, CCL2, TNF, EGFR
Positive regulation of ERK1 and ERK2 cascade	IL6, JUN, CCL2, TNF, EGFR, VEGFA
Inflammatory response	IL6, CXCL8, IL1B, CCL2, AKT1, TNF

**Table 5:** The results of molecular docking.

Component	Target (gene symbol/protein name)	PDB ID	CDOCKER_ENERGY (kcal/mol)
Quercetin	TP53/cellular tumor antigen p53	4BUZ	−39.4732
Quercetin	AKT1/RAC-alpha serine/threonine-protein kinase	1H10	−26.3244
Quercetin	IL6/interleukin-6	1ALU	−26.6531
Quercetin	TNF/tumor necrosis factor alpha	3IT8	−15.4083
Quercetin	VEGFA/vascular endothelial growth factor A	4GLU	−24.2995
Quercetin	JUN/transcription factor AP-1	5FV8	−26.5148
Quercetin	MAPK1/mitogen-activated protein kinase 1	2WAJ	−37.9661
Quercetin	MMP9/matrix metalloproteinase-9	2OW1	−39.5964
Quercetin	EGF/pro-epidermal growth factor	3NJP	−14.2012
Quercetin	IL1B/interleukin-1*β*	1IRA	−22.1756
Quercetin	MYC/Myc proto-oncogene protein	4Y7R	−26.1611
Quercetin	EGFR/epidermal growth factor receptor	2JIV	−40.9744
Quercetin	CXCL8/interleukin-8	6LFM	−24.7365
Quercetin	CASP3/caspase-3	3DEI	−33.4638
Quercetin	CCL2/C-C motif chemokine 2	4DN4	−32.0911
Formononetin	JUN/transcription factor AP-1	5FV8	−9.6172
Kaempferol	AKT1/RAC-alpha serine/threonine-protein kinase	1H10	−21.7786
Kaempferol	TNF/tumor necrosis factor-alpha	3IT8	−8.2117
Kaempferol	JUN/transcription factor AP-1	5FV8	−20.2562
Kaempferol	MAPK8/mitogen-activated protein kinase 8	1UKI	−26.8723
Kaempferol	CASP3/caspase-3	3DEI	−24.6836

**Table 6:** Summary of key targets' functional research.

Key target	Functional research in cisplatin-induced kidney injury	References
TNF-*α*	1. Cisplatin ⟶ TNF-*α* ⟶ TNFR1 ⟶ exogenous apoptotic pathway	[47]
	2. Cisplatin ⟶ TNF-*α* ⟶ NF-*κ*B ⟶ oxidative stress ⟶ kidney damage	[48]
	3. TNF-*α* ⟶ RIP1, RIP3, and MLKL ⟶ necrotic signaling pathway	[49]

p53	1. Cisplatin ⟶ DNA damage ⟶ p-p53 ⟶ Bax ⟶ Bcl-2 and Bcl-xL ⟶ mitochondrial apoptotic pathway	[47]
	2. Cisplatin ⟶ p53 ⟶ P66shc and MnSOD ⟶ oxidative damage and apoptosis	[51]
	3. AMPK-p53-Bax signaling pathway (Cisplatin ⟶ AMPK ⟶ p-p53 ⟶ Bax and caspase 3 ⟶ apoptosis)	[44]

IL-6	1. Cisplatin ⟶ IL-6 ⟶ inflammatory response ⟶ cisplatin-induced kidney injury	[52–55]
	2. IL-6 mediates the production and elimination of ROS in cisplatin-induced AKI	[56]

VEGFA	VEGFA ⟶ improve microcirculation and antiapoptotic ⟶ against cisplatin-induced AKI	[57]

EGF/EGFR	EGF-EGFR ⟶ tyrosine kinase ⟶ Ras ⟶ MAPK signaling pathway	[58]

MAPK1/MAPK8/JUN	MAPK signaling pathway	[54, 59–61]

IL-1*β*	IL-1*β* and IL-6 ⟶ neutrophil infiltration ⟶ cisplatin-induced AKI	[62]

CXCL8	Cisplatin ⟶ CXCL8 ⟶ neutrophil recruitment ⟶ inflammatory response ⟶ cisplatin-induced kidney injury	[63, 64]

Caspase-3	Cisplatin ⟶ TNFR1, TNFR2, and FasR ⟶ caspase-8 ⟶ caspase-3 ⟶ apoptosis	[43]

## Data Availability

Processed data are contained within the article. Raw data are available from the corresponding author upon request.

## Abbreviations

AM: *Astragalus membranaceus*
AKI: Acute kidney injury
C-T-P network: Network of active component-target-pathway
DC: Degree centrality
DL: Drug likeness
DS: Discovery Studio
EGFR: Epidermal growth factor receptor
GO: Gene Ontology
IL-1*β*: Interleukin-1*β*
IL-6: Interleukin-6
KEGG: Kyoto Encyclopedia of Genes and Genomes
MAPK1: Mitogen-activated protein kinase 1
MLKL: Mixed lineage kinase domain-like protein
MMP-9: Matrix metalloproteinase-9
MnSOD: Manganese-dependent superoxide dismutase
OB: Oral bioavailability
p53: Cellular tumor antigen p53
PDB: Protein Data Bank
PPI: Protein-protein interaction
RIP1: Receptor-interacting protein 1
TCMSP: Traditional Chinese Medicine Systems Pharmacology
TNFR1: Tumor necrosis factor receptor 1
TNF-*α*: Tumor necrosis factor-alpha
VEGFA: Vascular endothelial growth factor A.
